# Electrospun Conductive Composites with Anisotropic Microstructures and Tunable Mechanical Properties for Wearable Bioelectronics

**DOI:** 10.3390/ma19040684

**Published:** 2026-02-11

**Authors:** Jing Liu, Chang Liu, Ankang Du, Yiming Liu, Yunxiang Feng, Yujie Zhang, Zhifeng Pan, Lijun Lu, Yanchao Mao

**Affiliations:** Key Laboratory of Materials Physics of Ministry of Education, School of Physics, Zhengzhou University, Zhengzhou 450001, China; ljing_@gs.zzu.edu.cn (J.L.);

**Keywords:** electrospinning, anisotropy, ionogel, flexible electrode, physiological signal monitoring

## Abstract

This study seeks to resolve the critical yet often conflicting demands for electrical stability and mechanical tunability in flexible materials for wearable electronics. A composite conductive material was prepared based on the combination of electrospun fiber networks with tunable orientation and ion-gel phase. Through structural regulation, we achieved the designed adjustment of mechanical properties from isotropic to anisotropic while maintaining stable electrical conductivity. By adjusting the fiber orientation, Young’s modulus can be tailored to span a broad range. The fabricated composite membrane was processed into a flexible dry electrode and used for electrocardiogram (ECG) signal acquisition, achieving a high signal-to-noise ratio and stable waveform characteristics. Additionally, it can reliably monitor electromyographic signals from various static and dynamic hand gestures, including clenching, unclenching, and thumbs-up motions. This work provides a viable way to design materials and construct structures for multifunctional wearable electronic devices.

## 1. Introduction

The rapid advancement of flexible electronics is reshaping the landscape in multiple critical fields, particularly in frontier areas such as personalized health monitoring, intelligent human–machine interaction, and precision medicine [1,2,3,4,5,6,7]. Flexible sensors for next-generation wearables have now advanced to the point of achieving conformal skin integration and stable functionality even under significant mechanical deformation. A key challenge is how to create material systems that simultaneously meet the requirements for both excellent mechanical compliance and reliable electrical performance [8,9,10,11,12,13]. However, the design requirements for the mechanical and electrical properties of common materials frequently conflict. Enhancing electrical conductivity generally necessitates the incorporation of rigid functional fillers, which unavoidably compromise material flexibility and mechanical durability. Conversely, design techniques meant to maximize mechanical performance frequently have a negative impact on electrical stability. These intrinsic limitations act as a bottleneck, restricting the performance of flexible electronics in real-world applications and thereby hindering their growth [3,14,15,16,17,18,19,20,21,22,23,24].

Among the various emerging flexible material systems, the fiber membranes produced by electrospinning technology present unique potential for advanced applications. This advanced manufacturing technology generates three-dimensional fiber networks with programmable microstructures by precisely manipulating electric field parameters, solution properties, and collection methods. The resulting multi-level structure combines ideal high porosity with an ultra-large specific surface area, while also exhibiting excellent tunability in mechanical properties [25,26,27,28,29,30]. These characteristics collectively provide electrospun fiber membranes with dual advantages. The internal fibers form a stable three-dimensional network, acting like a robust skeleton that effectively disperses pressure and withstands external forces. Moreover, The combination of a porous structure and a high surface area facilitates the straightforward integration of diverse functional materials. The unique material properties make it an ideal platform for developing high-performance flexible electronic devices, offering a broad foundation for achieving multifunctional composite integration [31,32].

Notably, accurate modulation of key processing parameters, particularly the collection apparatus’s rotational speed, offers advanced control over the spatial alignment of electrospun fibers. This critical structural parameter exerts a decisive influence on the macroscopic mechanical behavior of the material. Mechanical anisotropy emerges and becomes more pronounced as the fiber orientation increases [33,34,35,36]. Meanwhile, ionogels, as a novel class of soft materials composed of ionic liquids and crosslinked polymer networks, have emerged as a promising candidate in the field of flexible electronics due to their unique physicochemical properties. The combination of high ionic conductivity, intrinsic flexibility, and stability across a broad temperature range renders ionogels a compelling substitute for traditional conductive materials [37,38,39,40,41,42,43,44,45,46,47,48,49,50]. Integrating electrospun fiber membranes and ionogels can result in a synergistic combination of their distinct benefits. The fibrous scaffold provides tunable mechanical support, and the ionogels aid in forming highly efficient conductive pathways. However, despite extensive research focusing on their individual properties, systematic investigations into their composite system remain scarce. Furthermore, the practical performance of this composite material in flexible electronic devices still lacks thorough evaluation [51,52].

Here, we fabricated poly(ethylene oxide) (PEO) electrospun fiber membranes with controlled alignment and systematically evaluated their performance upon integration with an ionogel. By precisely tuning the collector rotation speed (100, 500, and 2000 rpm), we achieved programmable microstructural control, transitioning the fiber morphology from randomly oriented to highly aligned. Mechanical testing establishes fiber orientation as a critical factor controlling both the overall mechanical behavior and the tensile strength of the material. Specifically, membranes produced at 100 rpm displayed isotropic properties with fracture stresses of approximately 1.5 MPa in both testing directions. In contrast, samples prepared at 500 rpm demonstrated 2.4 MPa fracture stress along the fiber direction, while only 1.3 MPa in the transverse orientation. The most pronounced anisotropy emerged in the 2000 rpm membranes, which achieved 7.0 MPa fracture stress parallel to the fiber alignment compared to merely 0.5 MPa in the perpendicular direction. In addition, all three composites maintained stable conductivity, and we validated the application potential of 2000 rpm composite membranes for physiological signal monitoring, demonstrating a superior electrocardiogram (ECG) signal-to-noise ratio (27 dB) over commercial electrodes (23 dB) and clear discrimination of multiple hand gestures through EMG signals. This work establishes a material design methodology that enables the development of high-performance flexible electronics with adjustable mechanical properties.

## 2. Materials and Methods

### 2.1. Materials

Poly(ethylene oxide) (PEO, Mn = 600,000), poly(ethylene glycol) diacrylate (PEGDA, Mn = 700), benzyl acrylate (BA), poly(ethylene glycol) methyl ether methacrylate (PEGMA, Mn = 950), and Sudan I were purchased from Macklin (Shanghai, China). 2,4,6-Trimethylbenzoyl diphenylphosphine oxide (TPO) and 1-ethyl-3-methylimidazolium dicyanamide ([EMIm][DCA]) were obtained from Aladdin. Deionized water was prepared using a laboratory ultrapure water system. All chemicals were utilized without any additional purification.

### 2.2. Fabrication of Electrospun Membranes

**Figure 1:**
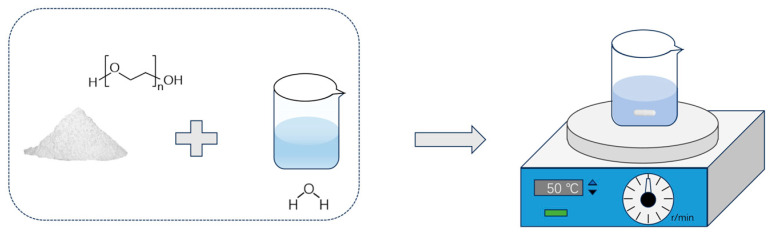
Schematic illustration of the preparation of the electrospinning precursor solution.

**Figure 2:**
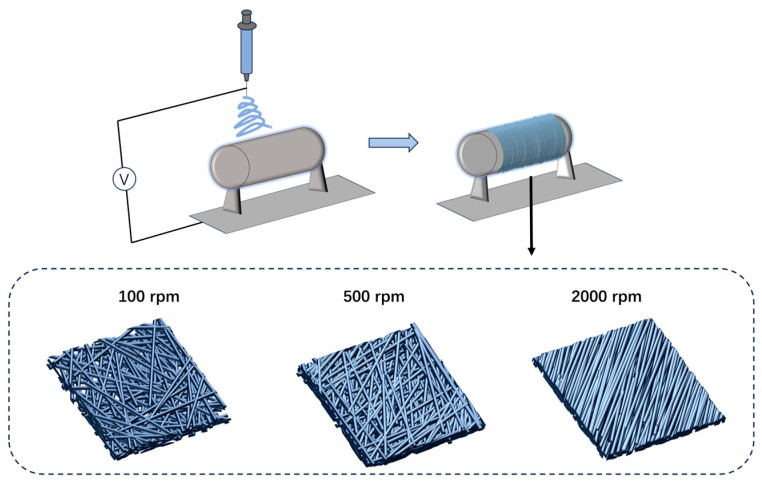
Schematic diagram of electrospun membranes prepared at different rotational speeds, illustrating differences in fiber alignment.

Poly(ethylene oxide) (PEO) fibrous membranes with controlled alignment were prepared using an electrospinning technique. First, PEO powder (4 wt%) was dissolved in deionized water under magnetic stirring at 50 °C for 3 h to form a homogeneous spinning dope. The prepared solution was then transferred to a plastic syringe fitted with a 21-gauge steel flat-tip needle. The electrospinning process was conducted under controlled environmental conditions, with the ambient temperature maintained at approximately 35 °C and the relative humidity at around 40%. A voltage of 15–20 kV was applied, and the distance between the needle tip and the rotating drum collector was set to 15–20 cm, as illustrated in Figure 1. To achieve different fiber alignment structures, the collector’s spinning speed was methodically adjusted at three levels: 100 rpm for randomly oriented membranes, 500 rpm for partially aligned membranes, and 2000 rpm for highly uniaxially aligned membranes (Figure 2).

### 2.3. Fabrication of Composite Conductive Membranes

The PEO/ionogel composite conductive membranes were prepared by integrating the electrospun fiber mats with a UV-curable ionogel precursor. The precursor solution was formulated by dissolving PEGDA (0.2 wt%), TPO (1 wt%), [EMIm][DCA] (10 wt%), and trace Sudan I in a BA/PEGMA (7:3) mixed solvent system. Stir the mixture until completely homogeneous and transparent. A suitable amount of this precursor solution is then evenly coated onto the surface of the PEO electrospun membrane. Upon sufficient penetration of the solution into the porous fiber network, the sample is exposed to 365 nm ultraviolet light for 5 min to induce crosslinking and cure the structure. A structurally stable PEO/ionogel composite conductive fiber membrane is ultimately obtained.

### 2.4. Characterization and Testing Methods

**Microstructural Characterization**. Surface morphology of electrospun fiber membranes was observed using a field emission scanning electron microscope (FE-SEM, ZEISS Sigma 360, Jena, Germany). To enhance surface conductivity, samples underwent gold sputter coating prior to imaging. Observations were conducted at an accelerating voltage of 3.0 kV. The obtained SEM images were statistically examined for fiber angles using ImageJ software (Windows x86-64, version dated 30 May 2017; National Institutes of Health, Rockville, MD, USA), and the fiber orientation distribution was quantitatively assessed using Fast Fourier Transform (FFT) analysis.

**Mechanical Characterization**. The tensile properties of the materials were characterized using a Mark-10 mechanical testing system (Mark, Irvine, CA, USA). Before testing, the samples were chopped into 15 mm × 5 mm rectangular strips and mounted on conventional tensile fixtures. All tests were carried out at room temperature, with samples stretched at a steady rate of 5 mm/min until fractured. The load–displacement data are automatically recorded by the system to generate an engineering stress–strain curve, where Young’s modulus is derived from the slope of the initial linear segment of the curve. All results are presented as mean ± standard deviation.

**Electrical Characterization**. The vertical conductivity of the material is measured with the ST-2258C four-point probe device. The sample is put on the instrument’s bottom plate, and the four probes are lowered to maintain stable contact with the sample surface. A constant DC current (I) is applied in the thickness direction of the sample, and the corresponding voltage drop (U) is measured. The vertical resistance (R) is then calculated using Ohm’s law:

R = U/I

The electrical conductivity (σ) is calculated using the following formula:

σ = L/RA

where L is the average sample thickness (cm), and A is the effective contact area between the probe and the sample (cm^2^). During testing, the system interface directly displays real-time conductivity data. The final results are reported as mean ± standard deviation.

**Physiological Signal Monitoring**. Electrocardiogram (ECG) signals were acquired using a Heart and Brain Spiker Box bioamplifier (Backyard Brains, Ann Arbor, MI, USA). Two composite membrane electrodes were attached to the subject’s left and right wrists, with the reference electrode placed on the dorsum of the right hand. Electromyogram (EMG) signals were recorded using a Muscle Spiker Shield Pro system (Backyard Brains, USA), where two recording electrodes were positioned along the target muscle group of the left forearm with a 2 cm inter-electrode spacing, and the reference electrode was similarly placed on the dorsum of the right hand. All physiological signals were simultaneously acquired at a sampling rate of 1 kHz, with real-time visualization and data storage accomplished through the manufacturer’s proprietary software. Commercial Ag/AgCl electrodes (3M Medical Devices, Shanghai, China) served as the reference in all electrophysiological tests.

## 3. Results and Discussion

### 3.1. Microstructural Analysis of Composite Membranes

**Figure 3:**
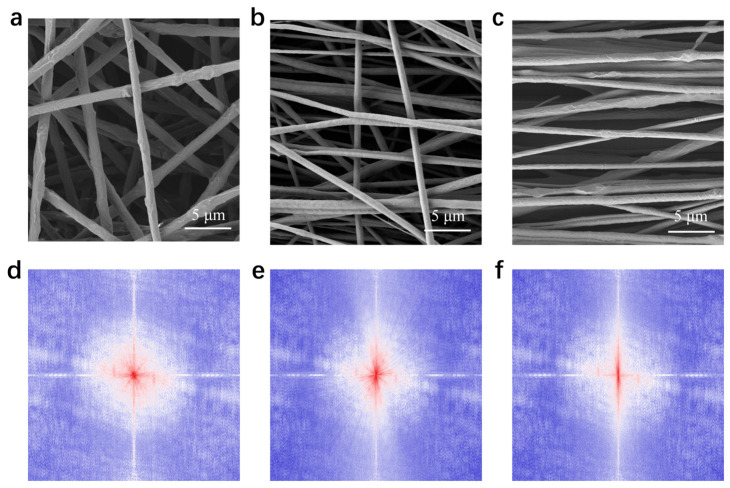
Morphology and orientation analysis of PEO electrospun fibers prepared at different collector rotational speeds with SEM images at (**a**) 100 rpm, (**b**) 500 rpm, and (**c**) 2000 rpm, along with corresponding FFT analysis patterns in (**d**–**f**).

We first achieved precise control and characterization of the microstructural orientation in the fiber scaffolds. Figure 3 presents the morphology and multilevel orientation analysis of PEO electrospun fiber membranes produced at varying collector rotational speeds. The results include typical scanning electron microscopy (SEM) images, fiber angle distribution histograms obtained through image processing, and fast Fourier transform (FFT) patterns. FFT analysis converts structural information from spatial images to the frequency domain, in which the dominant pattern direction remains perpendicular to the actual fiber orientation. Angular distribution statistics provide a quantitative analysis of fiber orientation [53,54,55,56].

**Figure 4:**
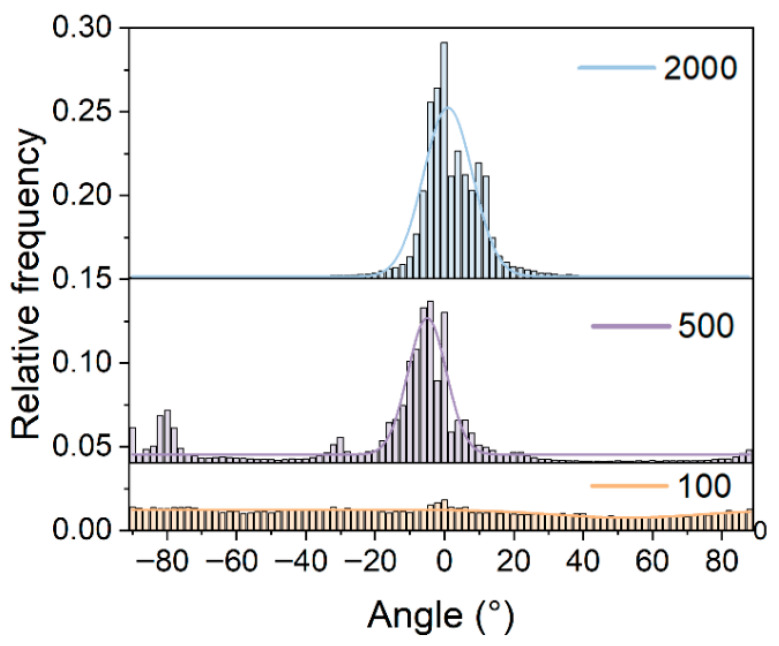
Comparative fiber orientation distributions at three rotational speeds: 100, 500, and 2000 rpm, respectively.

At the initial speed of 100 rpm, the fibers assemble into a random three-dimensional network, resulting in a completely disordered structure (Figure 3a). The corresponding FFT spectrum (Figure 3d) exhibits a uniform, continuous circular halo, demonstrating that image spatial frequencies are evenly distributed in all directions without showing any pronounced preferred orientation. This further validates the isotropic nature of the fiber structure. The random fiber morphology is well-supported by the angle distribution histogram (Figure 4). The histogram shows a broad, flat distribution across the entire −90° to 90° range, directly indicating the highly disordered arrangement of the fibers. This disordered morphology is primarily attributed to the dominant effects of random disturbances and Brownian motion during low-speed deposition.

At 500 rpm (Figure 3b), the fibers begin to align along the collector’s rotation direction (horizontal direction), though they still have multiple crossings and curvatures, indicating a transitional condition of imperfect alignment. This structural change is clearly reflected in the FFT image. Figure 3e indicates that the original uniform circular ring is warped and extended vertically, resulting in a brilliant and short linear feature. The perpendicular relationship between the vertical FFT signal and the horizontally aligned fibers observed by SEM reveals a dominant orientation and its corresponding spatial frequency distribution along the horizontal axis. Meanwhile, the angular distribution histogram (Figure 4) clearly shows that the fiber orientation distribution becomes concentrated and forms a large peak at 0°, indicating the formation of a preliminary but imperfect orientational order.

**Figure 5:**
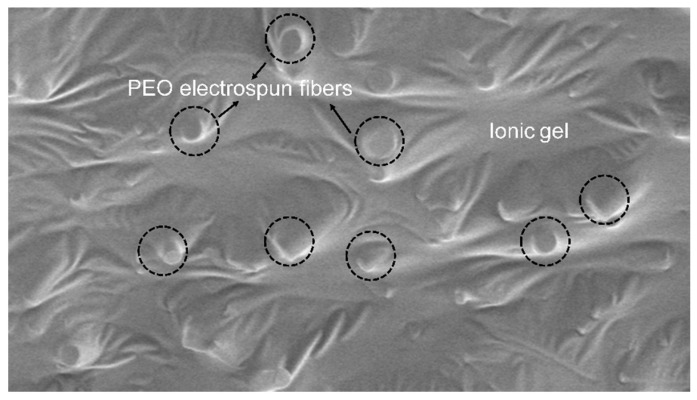
SEM image showing the the fractured cross-section of the composite film, consisting of an electrospun fiber membrane integrated with a uniform ionogel coating. The circle indicates the PEO electrospun fibers.

At 2000 rpm, SEM images (Figure 3c) reveal a highly uniform, nearly parallel fiber alignment with significantly reduced crossings and entanglements. The corresponding FFT image (Figure 3f) exhibits a distinct, high-contrast vertical line. The orientation of this vertical line (90°) is strictly perpendicular to the actual fiber orientation direction (0°). This unique FFT pattern is characteristic of highly ordered, unidirectionally aligned structures, indicating that the fiber array possesses a single, clearly dominant direction in spatial frequency. Finally, the angular distribution statistics (Figure 4) provide the most direct evidence of this special ordering. The corresponding distribution curve evolves into a sharply defined, high-intensity single peak, concentrated near 0°, clearly indicating that the fibers exhibit a high degree of orientation and good consistency. This highly ordered structure primarily arises from the synergistic effects of intense mechanical shear generated by high-speed rotation and the stretching action of the electric field, which, combined, promote the deposition of fibers in a highly oriented manner. The fractured cross-section of the fiber/gel composite film was characterized, as shown in Figure 5. After coating with the ion gel, the electrospun fibrous membrane presents a smooth and continuous surface while retaining its underlying network structure. This integrated architecture provides an ideal microstructure for ensuring stable interfacial contact and efficient ion transport in subsequent applications.

### 3.2. Mechanical Performance Analysis

**Figure 6:**
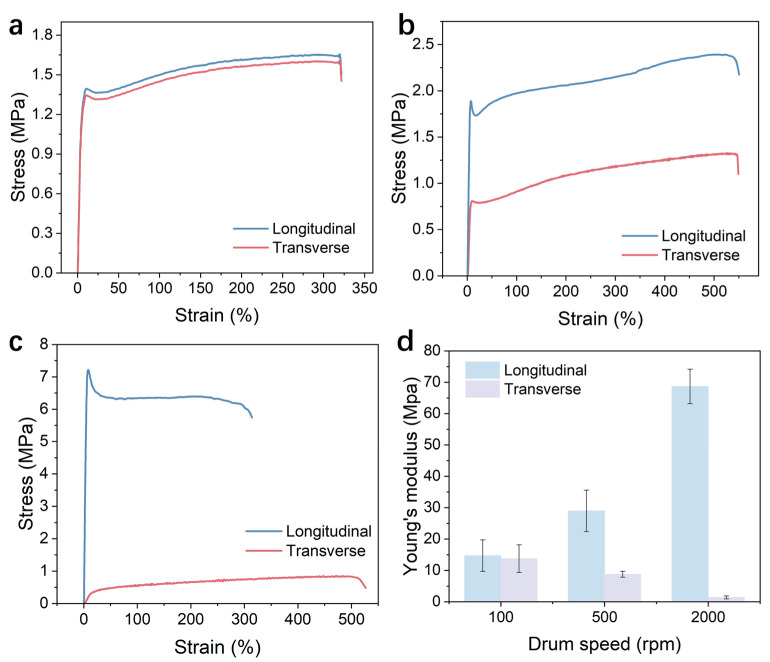
Mechanical anisotropy of electrospun membranes: (**a**–**c**) Representative stress–strain curves along (0°) and perpendicular (90°) to fiber direction at 100, 500, and 2000 rpm. (**d**) Young’s modulus statistics determined from initial linear regions.

Building on a well-defined microstructure, we further explored the critical role of fiber orientation in the macroscopic mechanical properties of fiber membranes. The influence of fiber alignment on mechanical properties was evaluated by performing tensile tests on PEO electrospun membranes prepared at varied rotational speeds. Measurements were taken both along the fiber orientation direction (0°) and perpendicular to it (90°). The test results strongly correlated with morphological analysis, demonstrating the material exhibits significant mechanical anisotropy through controlled fiber orientation. The stress–strain curves of PEO electrospun membranes prepared at 100 rpm are shown in Figure 6a. The curves along the spinning orientation and perpendicular to it are nearly identical, with only a slight difference in Young’s modulus (approximately 1.0 MPa), indicating pronounced mechanical isotropy. This result is consistent with the conclusions drawn from the above microstructural analysis. When the rotational speed increased to 500 rpm, the fibers exhibited a certain degree of orientation compared to the 100 rpm condition, and the corresponding stress–strain curves in the two directions began to diverge (Figure 6b). Along the fiber orientation direction (0°), the curve slope and maximum fracture stress were both higher than in the perpendicular direction (90°). The difference in Young’s modulus between the two directions was approximately 20 MPa, while the maximum fracture stress differed by about 1 MPa. This difference primarily stems from the more efficient load-bearing and transfer capabilities of fibers oriented along the direction of alignment, where mechanical responses in the perpendicular direction are more significantly influenced by weaker fiber-fiber interactions and slip behavior. The highly ordered PEO electrospun membranes showed noticeable variations in stress–strain behavior between the two testing directions when the rotation speed was raised to 2000 rpm (Figure 6c). Along the fiber orientation direction, the Young’s modulus can reach 74.0 MPa, while in the perpendicular direction it is only about 1 MPa. The difference between the two is approximately 73 MPa, approximately 74-fold. Additionally, the biggest tensile strength differential grew to about 6.8 MPa. The results reveal that in highly aligned constructions, the load is mostly carried by fiber bundles along the primary axis, while the mechanical response in the transverse direction is limited by weak van der Waals bonding between fibers, resulting in a direction of mechanical weakness. Young’s modulus data (Figure 6d) are displayed as bar charts and clearly show the controlled transition from isotropic to highly anisotropic mechanical behavior. For comparison, we summarize the typical ranges of Young’s modulus for commonly reported electrospun polymer membranes in Table 1. As shown, the Young’s modulus of the PEO membrane in this work can be tuned over a relatively wide range by controlling fiber orientation, enabling a good balance between flexibility and mechanical strength [57,58,59,60,61,62].

The mechanical properties of the fibrous membranes show a clear correlation with their microscopic orientation structure. By accurately manipulating the rotation speed of the electrospinning collector, the fiber arrangement was controlled from disordered to ordered, producing membranes that transition from mechanical isotropy to noticeable anisotropy. This tunable behavior endows the materials with strong potential for applications requiring directional load bearing or controlled deformation. A comparative analysis of representative studies in this field (Table 2) reveals that while existing fiber-based composites often pursue single performance extremes, the programmable fiber-alignment strategy presented here offers a distinctive route to achieving broadly tunable mechanical properties.

### 3.3. Electrical Performance Analysis

**Figure 7:**
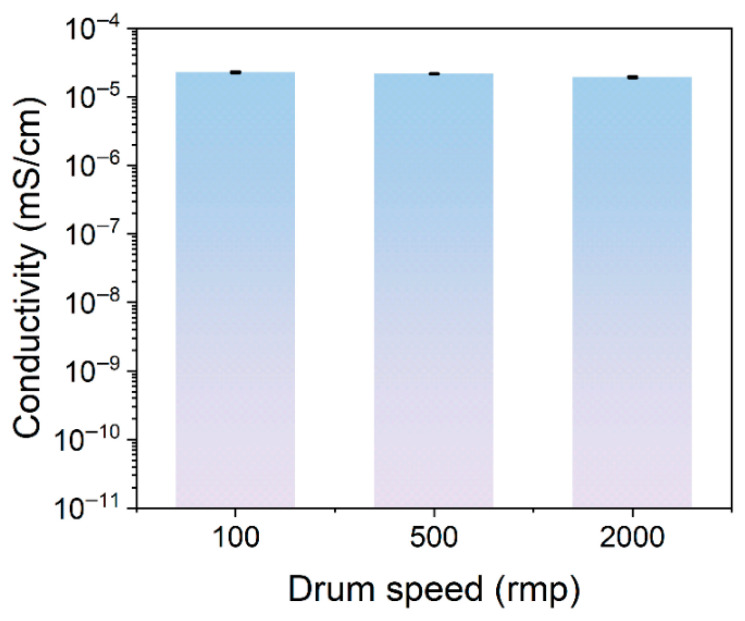
Electrical conductivity of ionogel composites based on PEO fibrous scaffolds with different alignment.

Compared to significant changes in mechanical properties, the electrical conductivity of composite materials exhibits good stability under structural variations. To build functional conductive composites, we selected electrospun PEO fiber membranes with different orientations as porous scaffolds and prepared composite systems by impregnating them with high-conductivity ionogel. To elucidate the impact of scaffold microstructure on composite conductivity, we systematically investigated how microstructural characteristics govern macroscopic electrical performance. All of the composites exhibited high and stable electrical conductivities (approximately 10^−5^ mS/cm), as shown in Figure 7. The main reason for this is the continuous distribution of the ionogel conductive phase within the materials. Composites prepared using fiber scaffolds at 100 rpm, 500 rpm, and 2000 rpm exhibited conductivities of 2.28 × 10^−5^, 2.17 × 10^−5^, and 1.94 × 10^−5^ mS/cm, respectively. In contrast to the significant changes in fiber orientation, the conductivity of the composites showed only a minor decline with increasing collector speed and remained within the same order of magnitude. The overall variation range was extremely limited, demonstrating that the fiber orientation structure regulated by collector speed is not the primary factor determining conductivity in this system. This observation contrasts with the pronounced mechanical anisotropy discussed in Section 3.2, indicating that the orientation of the fibrous scaffold predominantly affects the in-plane mechanical response of the composite, whereas its electrical performance is mainly governed by the infiltrated ion-gel phase.

### 3.4. Electrode-Skin Interface Impedance Characteristics

**Figure 8:**
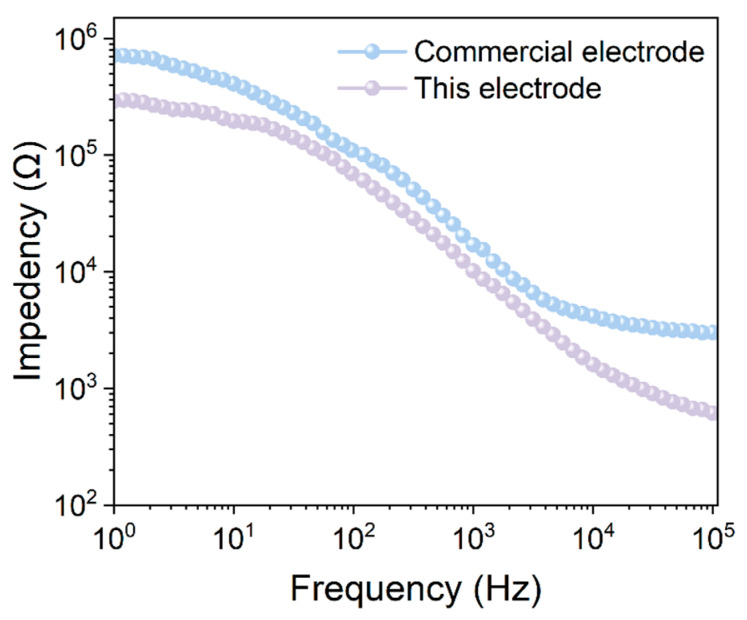
Interfacial impedance comparison between commercial and composite membrane electrodes.

The electrode-skin interface impedance critically determines the quality of acquired biopotential signals [63,64,65]. To evaluate the interfacial electrical properties of the composite electrode, we employed electrochemical impedance spectroscopy (EIS) to compare the impedance characteristics of the composite membrane electrode with the commercial electrode across the frequency range of 1–10^5^ Hz (Figure 8). A consistently lower impedance was observed for the composite membrane electrode compared to the commercial counterpart across all frequencies, confirming its reduced interfacial resistance. This phenomenon is closely related to the structural characteristics of the material. The ionogel phase forms continuous transport pathways within the fiber network, facilitating charge migration within the electrode. Concurrently, the multi-level pore structure provided by the fiber scaffold promotes the permeation and distribution of the electrolyte at the interface. The composite electrode’s consistent low-impedance behavior indicates the material can form an effective electrode-skin electrical contact, which is useful for reliable bioelectrical signal transmission. For the acquisition of weak physiological signals, such as electrocardiogram signals, as well as high-frequency electromyographic signals, a lower interfacial impedance helps to reduce signal attenuation and phase distortion, thereby improving signal quality. From the perspective of interfacial electrical properties, these results demonstrate the practical advantages of the composite membrane electrode and support its potential application in wearable health-monitoring systems.

**Table 2:** Comparison of material strategies, performance, and applications in fiber-based wearable bioelectronics.

Dimension	Material System	Structural Design Strategy	Mechanical Performance	Electrical Performance	Demonstrated Application
MXene Composite Fibers (2025) [66]	MXene, CNTs, PLA	Static-dynamic densification	Ultrahigh strength (941.5 MPa)	High electronic conductivity	Wireless e-textiles
Breathable Textile Electrodes (2026) [67]	Cotton, MXene, PEDOT:PSS	Surface functionalization coating	Intrinsic fabric flexibility	Stable for biopotentials	ECG, EMG, EEG monitoring
TPEE/PPy Membrane (2026) [68]	TPEE fibers, PPy, TA	Electrospun substrate in situ polymerization	High stretchability (0.4–1.3 Mpa)	Moderate electronic conductivity	AI diagnosis
WADE-Skin (2023) [69]	SBS/PAAND fibers, EGaIn	Multilayer fibrous stack	Skin-like softness (850 kPa)	Low impedance	ECG and HMI
PEO/Ionogel Membranes (This Study)	PEO fibers, ([EMIm][DCA])	Programmable fiber alignment	Programmable anisotropy (1–74 MPa)	Stable ionic conductivity	bioelectronic interfaces for ECG and EMG

### 3.5. Physiological Electrical Signal Monitoring Application

**Figure 9:**
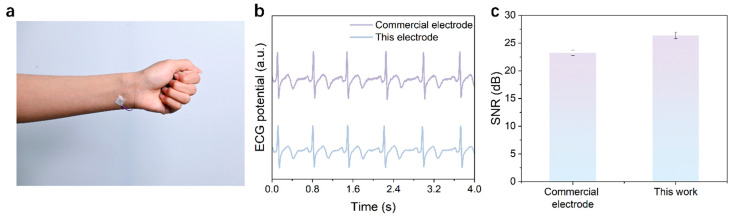
ECG monitoring performance. (**a**) Photograph of the composite membrane electrode recording ECG signals. (**b**) Simultaneously recorded ECG signals (upper: commercial gel electrode, lower: composite membrane electrode). (**c**) Corresponding signal-to-noise ratio statistics.

**Figure 10:**
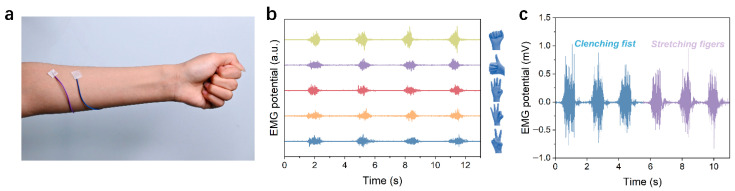
EMG monitoring performance. (**a**) Photograph of the composite membrane electrode acquiring EMG signals. (**b**) EMG signals of five hand gestures (victory, three, four, thumbs-up, fist) recorded with composite membrane electrodes. (**c**) Dynamic EMG responses during repetitive fist clenching and releasing cycles.

For validating the performance of the prepared highly oriented composite fiber membrane (2000 rpm) in wearable bioelectronic applications, we fabricated the material into flexible dry electrodes and evaluated their performance against commercial gel electrodes in monitoring typical physiological signals, specifically ECG and electromyogram (EMG). The performance of the electrodes was initially evaluated under static, low-amplitude ECG signal acquisition conditions. The electrodes were applied to the radial artery locations on both wrists of the test subjects (Figure 9a). Test results demonstrated that the composite membrane electrode produced a more stable ECG signal baseline and lower noise levels, allowing clear identification of characteristic waveforms such as P waves and T waves (Figure 9b). In contrast, the commercially available gel electrode exhibited noticeable baseline drift and high-frequency noise under identical testing conditions. This difference was further shown in quantitative analysis. The composite membrane electrode achieved an average signal-to-noise ratio (SNR) of 27 dB, higher than the 23 dB SNR of the commercial electrode (Figure 9c). These results demonstrate that the composite membrane electrode achieves a more stable and noise-resistant skin interface than conventional alternatives. This property is critical for reliably acquiring weak cardiac signals, such as those from the wrist. The performance of the electrode was further evaluated under dynamic, high-amplitude EMG recording conditions. The electrode was attached to the belly of the forearm muscle for testing (Figure 10a), where it enabled stable acquisition of EMG signals corresponding to five different hand gestures, each exhibiting clearly distinguishable features in the recorded EMG traces (Figure 10b). The signals associated with different gestures showed pronounced differences in amplitude and temporal evolution, reflecting the electrode’s effective response to the spatial distribution of muscle activity. During repeated fist clenching and relaxation tests (Figure 10c), the electrode exhibited consistent responses and mechanical stability. The signal was characterized by rapid burst peaks during contraction and equally rapid baseline recovery during relaxation, reflecting excellent dynamic response characteristics. These findings suggest the composite electrode is well adapted for EMG signal capture under dynamic situations, which may give experimental support for its use in wearable physiological monitoring.

## 4. Conclusions

In summary, this work developed a conductive composite material system consisting of an ionogel embedded within an electrospun fiber scaffold with structurally tunable anisotropy. The scaffold morphology was controlled from random to highly aligned by simply changing the electrospinning collector speed, enabling a wide and predictable modulation of the mechanical properties of the electrospun fiber scaffold (e.g., a modulus spanning 1–74 MPa). Such a level of tunability is rarely achieved in conventional fiber-based composites. Meanwhile, the composites exhibited relatively stable conductivity under different orientation conditions, primarily attributed to the conductive pathways provided by the continuously distributed ionogel phase within the fiber network. The tunable mechanical properties coupled with stable electrical performance render these composites suitable for fabricating flexible dry electrodes for reliable bioelectrical signal monitoring. It achieves a higher signal-to-noise ratio in ECG monitoring (27 dB versus 23 dB) compared to commercial electrodes and enables clear discrimination and stable tracking of multiple hand gestures in EMG detection. This work not only provides a novel material platform with excellent performance but also confirms that precise mechanical property control can be achieved through fiber orientation regulation. These findings validate its practical value for wearable health monitoring and bioelectronics.

## Figures and Tables

**Table 1:** Summary of the typical Young’s modulus ranges for common electrospun polymer membranes.

Polymer	PEO	PCL	PVA	TPU	PVDF	PAN
Young’s modulus	1–74 MPa	3–5 MPa	1–10 MPa	1–10 MPa	3–15 MPa	5.7–9.4 MPa

## Data Availability

The original contributions presented in the study are included in the article. Further inquiries can be directed to the corresponding authors.

## References

[1] Chen X.G., Wang C.H., Wei W., Liu Y.H., Ge S.S., Zhou L.Q., Kong H. (2025). Flexible and sensitive pressure sensor with enhanced breathability for advanced wearable health monitoring. npj Flex. Electron..

[2] Wang Y.Q., Sun C.C., Ahmed D. (2025). A smart acoustic textile for health monitoring. Nat. Electron..

[3] Yang M., Cheng Y.F., Yue Y., Chen Y., Gao H., Li L., Cai B., Liu W.J., Wang Z.Y., Guo H.Z. (2022). High-performance flexible pressure sensor with a self-healing function for tactile feedback. Adv. Sci..

[4] Liu D.J., Zhu P.C., Zhang F.K., Li P.S., Huang W.H., Li C., Han N.N., Mu S.R., Zhou H., Mao Y.C. (2023). Intrinsically stretchable polymer semiconductor based electronic skin for multiple perceptions of force, temperature, and visible light. Nano Res..

[5] Lu L.J., Wu J., Zhang Y.J., Liu C., Hu Y., Chen B.J., Zhu Y., Mao Y.C. (2025). Noncontact 3D gesture recognition enabled VR human-machine interface via electret-nanofiber-based triboelectric sensor. Nano Res..

[6] Zhang W., Lou Q., Sun J.L., Liao J., Zheng G.S., Jiao F.H., Chen W., Li X., Meng J.J., Shan C.X. (2025). Carbon nanodot-based flexible and self-powered white displays. Nano Res..

[7] Zhu P.C., Zhang B.S., Wang H.Y., Wu Y.H., Cao H.J., He L.B., Li C.Y., Luo X.P., Li X., Mao Y.C. (2022). 3D printed triboelectric nanogenerator as self-powered human-machine interactive sensor for breathing-based language expression. Nano Res..

[8] Xu B.Y., Yang M.Y., Cheng W.J., Li X.Y., Xu X.M., Li W.M., Zhang H., Zhou M. (2025). Precision aerosol-jet micropatterning of liquid metal for high-performance flexible strain sensors. Nat. Commun..

[9] Feng T.X., Ling D., Li C.Y., Zheng W.T., Zhang S.C., Li C., Emel’yanov A., Pozdnyakov A.S., Lu L.J., Mao Y.C. (2024). Stretchable on-skin touchless screen sensor enabled by ionic hydrogel. Nano Res..

[10] Chen X.X., Yang X., Lou Q., Zhang Y., Chen Y.C., Lu Y.C., Dong L., Shan C.X. (2022). Fabry-Perot interference and piezo-phototronic effect enhanced flexible MoS2 photodetector. Nano Res..

[11] Zhu P.C., Mu S.R., Huang W.H., Sun Z.Y., Lin Y.Y., Chen K., Pan Z.F., Haghighi M.G., Sedghi R., Wang J.L. (2024). Soft multifunctional neurological electronic skin through intrinsically stretchable synaptic transistor. Nano Res..

[12] Zhu P.C., Niu M.J., Liang S.Y., Yang W.Q., Zhang Y.T., Chen K., Pan Z.F., Mao Y.C. (2025). Non-hand-worn, load-free VR hand rehabilitation system assisted by deep learning based on ionic hydrogel. Nano Res..

[13] Chen B., Yu R., Wang J., Feng Y., Zhang Y., Mao Y., Shan C., Wang X. (2025). Biomaterials-Based Hydrogel with Superior Bio-Mimetic Ionic Conductivity and Tissue-Matching Softness for Bioelectronics. Adv. Funct. Mater..

[14] Gao M.Y., Liu W.J., Chen K., Sun H.L., Liu X.Q., Xing H.N., Wang H.T., Zhu B.P., Guo H.Z. (2025). Piezoresistive Effect: A New Concept for Hearing Aids. Adv. Sci..

[15] Han S.J., Liu C.R., Xu H.H., Yao D.Y., Yan K.H., Zheng H.L., Chen H.J., Gui X.C., Chu S., Liu C. (2018). Multiscale nanowire-microfluidic hybrid strain sensors with high sensitivity and stretchability. npj Flex. Electron..

[16] Li W.W., Yang S., Shamim A. (2019). Screen printing of silver nanowires: Balancing conductivity with transparency while maintaining flexibility and stretchability. npj Flex. Electron..

[17] Lu L.J., Hu G.S., Liu J.Q., Yang B. (2024). 5G NB-IoT System Integrated with High-Performance Fiber Sensor Inspired by Cirrus and Spider Structures. Adv. Sci..

[18] Matsuhisa N., Inoue D., Zalar P., Jin H., Matsuba Y., Itoh A., Yokota T., Hashizume D., Someya T. (2017). Printable elastic conductors by in situ formation of silver nanoparticles from silver flakes. Nat. Mater..

[19] Matsuhisa N., Kaltenbrunner M., Yokota T., Jinno H., Kuribara K., Sekitani T., Someya T. (2015). Printable elastic conductors with a high conductivity for electronic textile applications. Nat. Commun..

[20] Park J., Wang S.D., Li M., Ahn C., Hyun J.K., Kim D.S., Kim D.K., Rogers J.A., Huang Y.G., Jeon S. (2012). Three-dimensional nanonetworks for giant stretchability in dielectrics and conductors. Nat. Commun..

[21] Sunwoo S.H., Kim H.J., Kim J.H., Kim D.C., Kim D.H. (2025). Intrinsically soft electronics using conducting nanomaterials and liquid metals. NPG Asia Mater..

[22] Tan P., Wang H.F., Xiao F.R., Lu X., Shang W.H., Deng X.B., Song H.F., Xu Z.Y., Cao J.F., Gan T.S. (2022). Solution-processable, soft, self-adhesive, and conductive polymer composites for soft electronics. Nat. Commun..

[23] Xie Z.L., Zhu J.N., Dou Z.L., Zhang Y.Z., Wang K., Wu K., Fu Q. (2024). Liquid metal interface mechanochemistry disentangles energy density and biaxial stretchability tradeoff in composite capacitor film. Nat. Commun..

[24] Xu Q.G., Chu N.N., Wang Y., Wang H., Xu T.T., Li X.L., Huang S.Z., Li X.J., Luo Y.S., Yang H.Y. (2025). 3D Printed Low-Tortuosity and Ultra-Thick Hierarchical Porous Electrodes for High-Performance Wearable Quasi-Solid-State Zn-VOH Batteries. Adv. Sci..

[25] Chang S.L., Deng Y., Li N., Wang L.J., Shan C.X., Dong L. (2023). Continuous synthesis of ultra-fine fiber for wearable mechanoluminescent textile. Nano Res..

[26] Fatahian R., Erfani R. (2025). Surrogate modeling of electrospun PVA/PLA nanofibers using artificial neural network for biomedical applications. Sci. Rep..

[27] Peng X., Dong K., Ye C.Y., Jiang Y., Zhai S.Y., Cheng R.W., Liu D., Gao X.P., Wang J., Wang Z.L. (2020). A breathable, biodegradable, antibacterial, and self-powered electronic skin based on all-nanofiber triboelectric nanogenerators. Sci. Adv..

[28] Samadian H., Zamiri S., Ehterami A., Farzamfar S., Vaez A., Khastar H., Alam M., Ai A., Derakhshankhah H., Allahyari Z. (2020). Electrospun cellulose acetate/gelatin nanofibrous wound dressing containing berberine for diabetic foot ulcer healing: In vitro and in vivo studies. Sci. Rep..

[29] Smoak M.M., Hogan K.J., Grande-Allen K.J., Mikos A.G. (2021). Bioinspired electrospun dECM scaffolds guide cell growth and control the formation of myotubes. Sci. Adv..

[30] Sohrabi M., Razbin M. (2025). Hybrid modeling for optimizing electrospun polyurethane nanofibrous membranes in air filtration applications. Sci. Rep..

[31] Gilmore T.S., Gouma P.I. (2024). Scalable electrospinning using a desktop, high throughput, self-contained system. Sci. Rep..

[32] Hosseinian H., Jimenez-Moreno M., Sher M., Rodriguez-Garcia A., Martinez-Chapa S.O., Hosseini S. (2023). An origami-based technique for simple, effective and inexpensive fabrication of highly aligned far-field electrospun fibers. Sci. Rep..

[33] Persano L., Dagdeviren C., Su Y.W., Zhang Y.H., Girardo S., Pisignano D., Huang Y.G., Rogers J.A. (2013). High performance piezoelectric devices based on aligned arrays of nanofibers of poly(vinylidenefluoride-co-trifluoroethylene). Nat. Commun..

[34] Shaker A., Khedewy A.T., Hassan M.A., Abd El-Baky M.A. (2023). Thermo-mechanical characterization of electrospun polyurethane/carbon-nanotubes nanofibers: A comparative study. Sci. Rep..

[35] Tsuboi K., Marcelletti E., Matsumoto H., Ashizawa M., Minagawa M., Furuya H., Tanioka A., Abe A. (2012). Preparation of poly(γ-benzyl-L-glutamate) nanofibers by electrospinning from isotropic and biphasic liquid crystal solutions. Polym. J..

[36] Yu H.Q., Li Y., Song Y., Wu Y.B., Lan X.J., Liu S.M., Tang Y., Xu S.S., Chen B.J. (2017). Ultralong well-aligned TiO2: Ln3+ (Ln = Eu, Sm, or Er) fibres prepared by modified electrospinning and their temperature-dependent luminescence. Sci. Rep..

[37] Bu X.M., Ge Y.X., Wang L., Wu L.L., Ma X.F., Lu D.Y. (2021). Design of highly stretchable deep eutectic solvent-based ionic gel electrolyte with high ionic conductivity by the addition of zwitterion ion dissociators for flexible supercapacitor. Polym. Eng. Sci..

[38] Choi S.E., Oh S.J., Yoon J.M., Bae J.W. (2025). A Non-Volatile, Low-Voltage, Stretchable Transparent Dielectric Heater for Real-World Autonomous Thermal Management Platform. Small.

[39] He X.N., Zhang B., Liu Q.J., Chen H., Cheng J.X., Jian B.C., Yin H.L., Li H.G., Duan K., Zhang J.W. (2024). Highly conductive and stretchable nanostructured ionogels for 3D printing capacitive sensors with superior performance. Nat. Commun..

[40] Jung J., Lee S., Kim H., Lee W., Chong J., You I., Kang J. (2024). Self-healing electronic skin with high fracture strength and toughness. Nat. Commun..

[41] Lyu X.L., Yu K., Zhang H.Q., Zhou P.P., Shen Z.H., Zou Z.G. (2025). Tough fiber-reinforced composite ionogels with crack resistance surpassing metals. Nat. Commun..

[42] Marsavelski A., Smrecki V., Vianello R., Zinic M., Mogus-Milankovic A., Santic A. (2015). Supramolecular Ionic-Liquid Gels with High Ionic Conductivity. Chem.–Eur. J..

[43] Sun L., Feng W.W., Liu Y.C., Chen L.L., Chen T., Jin Z.K., Wang C. (2025). Elastic and ultra stable ionic conductors for long-life-time soft robots working at extreme environments. Nat. Commun..

[44] Wang H., Gupta A., Lu Q.C., Wu W.T., Wang X.Y., Huang X., Hu X.Y., Lee P.S. (2025). Ultrasoft and fast self-healing poly(ionic liquid) electrode for dielectric elastomer actuators. Nat. Commun..

[45] Wang J.Q., Wu B.H., Wei P., Sun S.T., Wu P.Y. (2022). Fatigue-free artificial ionic skin toughened by self-healable elastic nanomesh. Nat. Commun..

[46] Wang S.H., Liu H.Y., Yu Z.Y., Ren X.L., Hua Q., Panahi-Sarmad M., Yang P., Liu C.H., Renneckar S., Liu H. (2025). Cellulose-mediated ionic liquid crystallization enables tough-stiff switchable ionogels. Nat. Commun..

[47] Wang Y., Wei Z.X., Ji T.T., Bai R.B., Zhu H.L. (2024). Highly Ionic Conductive, Stretchable, and Tough Ionogel for Flexible Solid-State Supercapacitor. Small.

[48] Wen J.Q., Zhou L., Ye T.L. (2024). Polymer ionogels and their application in flexible ionic devices. SmartMat.

[49] Ye Z.W., Yu H.M., Xie H.X., Zhu W.W., Shi S.T., Liu C.C., Wang Y.Y., Liao J.Q., Sun Q.F., Zhao D.W. (2025). Flame-Retardant Ionogel Enabled by Lignin Molecular Networks for Fire Rescue. Adv. Sci..

[50] Zhou X., Zhou K.J., Tang L., Chen Z.Y., Hu Q.Y., Gao J., Zhang Y., Zhang J., Zhang S.G. (2024). A Strong and Highly Transparent Ionogel Electrolyte Enabled by In Situ Polymerization-Induced Microphase Separation for High-Performance Electrochromic Devices. Macromol. Rapid Commun..

[51] Lv B., Zhao G.R., Wang H.G., Wang Q.J., Yang B.P., Ma W., Li Z.Y., Li J.J. (2023). Ionogel Fiber-Based Flexible Sensor for Friction Sensing. Adv. Mater. Technol..

[52] Wang X., Gao Q.S., Schubert D.W., Liu X.H. (2023). Review on Electrospun Conductive Polymer Composites Strain Sensors. Adv. Mater. Technol..

[53] Meinhold K.L., Tankersley T., Darlington R., Robinson J.L. (2025). Mandrel Diameter Is a Dominating Parameter for Fiber Alignment Control in Rotating Mandrel Electrospinning Systems. J. Appl. Polym. Sci..

[54] Mondragón M., Arias E., Elizalde L.E., Castañeda M.E., Moggio I. (2017). Luminescence properties of aligned-electrospun fibers of poly(9-vinylcarbazole) doped with a europium (III) complex. J. Lumin..

[55] Sakamoto H., Asakawa H., Fukuma T., Fujita S., Suye S. (2014). Atomic force microscopy visualization of hard segment alignment in stretched polyurethane nanofibers prepared by electrospinning. Sci. Technol. Adv. Mater..

[56] Wang H.B., Mullins M.E., Cregg J.M., McCarthy C.W., Gilbert R.J. (2010). Varying the diameter of aligned electrospun fibers alters neurite outgrowth and Schwann cell migration. Acta Biomater..

[57] Croisier F., Duwez A.S., Jérôme C., Léonard A.F., van der Werf K.O., Dijkstra P.J., Bennink M.L. (2012). Mechanical testing of electrospun PCL fibers. Acta Biomater..

[58] Pouladzadeh F., Katbab A.A., Haghighipour N., Kashi E. (2018). Carbon nanotube loaded electrospun scaffolds based on thermoplastic urethane (TPU) with enhanced proliferation and neural differentiation of rat mesenchymal stem cells: The role of state of electrical conductivity. Eur. Polym. J..

[59] Stachewicz U., Bailey R.J., Wang W., Barber A.H. (2012). Size dependent mechanical properties of electrospun polymer fibers from a composite structure. Polymer.

[60] Sukiman M.S., Andriyana A., Ang B.C., Metselaar H.S.C. (2020). Elastic properties of electrospun PVDF nanofibrous membranes: Experimental investigation and numerical modelling using pixel-based finite element method. Polym. Test..

[61] Zaarour B., Zhu L., Huang C., Jin X. (2019). Enhanced piezoelectric properties of randomly oriented and aligned electrospun PVDF fibers by regulating the surface morphology. J. Appl. Polym. Sci..

[62] Huang L.W., Bui N.N., Manickam S.S., McCutcheon J.R. (2011). Controlling Electrospun Nanofiber Morphology and Mechanical Properties Using Humidity. J. Polym. Sci. B Polym. Phys..

[63] Sousa A.S.P., Noites A., Vilarinho R., Santos R. (2023). Long-Term Electrode-Skin Impedance Variation for Electromyographic Measurements. Sensors.

[64] Zhang Y.Z., Zhou J.L., Yang H.Y., Liu Q.X., Wang M., Xiong F., Chen D.Y., Du L.X. (2023). Effect of Fabric Electrode Surface Coating Medium on ECG Signal Quality under Dynamic and Static Conditions. Coatings.

[65] Ni Q.C., Lou Q., Shen C.L., Zheng G.S., Song R.W., Hao J.N., Liu J.L., Zhu J.Y., Zang J.H., Dong L. (2024). Sensitive humidity sensor based on moisture-driven energy generation. Nano Res..

[66] Zhou T., Yan J., Cao C., He Q., Li W., Chen L., Wu C., Feng Y., Lau D., Cheng Q. (2025). Ultrastrong MXene composite fibers through static-dynamic densification for wireless electronic textiles. Nat. Commun..

[67] Xu Z., Guo H., Cai J., Wang F., Li J., Tang Q., Yu J., Ding B., Li Z. (2026). Breathable and reusable fabric epidermal electrodes for personal health monitoring. Nano Energy.

[68] Liu J., Yi J., Shen W., Hu J., Fu T., Jing M., Qian H., Chen J., Li M., Lin Y. (2026). Highly Stretchable and Durable Thermoplastic Poly(ether–ester) Fibrous Membrane for Constructing a Deep Learning-Assisted Knee Deformity Diagnosis System. Adv. Fiber Mater..

[69] Chen F., Zhuang Q., Ding Y., Zhang C., Song X., Chen Z., Zhang Y., Mei Q., Zhao X., Huang Q. (2023). Wet-Adaptive Electronic Skin. Adv. Mater..

